# Rapid synthesis of azoindolizine derivatives via aryldiazonium salts

**DOI:** 10.55730/1300-0527.3675

**Published:** 2024-03-11

**Authors:** Ferruh LAFZI

**Affiliations:** Department of Chemistry, Faculty of Science, Atatürk University, Erzurum, Turkiye

**Keywords:** Indolizine, aryldiazonium, azobenzene, azoindolizine

## Abstract

A practical, rapid, and efficient method for the synthesis of azoindolizine derivatives via aryldiazonium salts with excellent yields was reported. Firstly, the corresponding aniline derivatives were synthesized via a simple and rapid method. Then, the optimal reaction conditions were investigated using a variety of protic and aprotic solvents that demonstrating the robustness of the approach. Finally, the applicability of this method to various sources of indolizine and phenyldiazonium tetrafluoroborate salts was expanded.

## 1. Introduction

Azobenzenes, as quintessential molecules, play a central role in both fundamental and applied research. Their journey spans nearly two centuries, witnessing remarkable accomplishments. Initially mere dyes, they have now transformed into versatile ‘little engines’, permeating various facets of our lives. From cosmetics, textiles, chemosensors, food, and medicine to photonics and energy, azobenzenes have become ubiquitous and impactful [1–8]. Even with their extensive history, azobenzenes remain a subject of academic fascination and are actively produced for industrial applications. This enduring interest can be attributed to their diverse chemistry, easy and adaptable design, reliable photoswitching capabilities, and eco-friendliness. The advancement of azobenzenes has led to the creation of novel colored and light-responsive materials, finding utility across various domains. As research progresses, their implementation in cutting-edge high-tech applications continues to expand [9–12]. Consequently, the synthesis of azobenzene derivatives with diverse structures, especially those connected to heterocycles, holds great significance [13–17]. Recent endeavors have focused extensively on creating bioactive heterocycles containing azo chromophores, as they display distinct properties stemming from effective conjugation and the electronic effects of substituents [18–22].

**Scheme 1:**
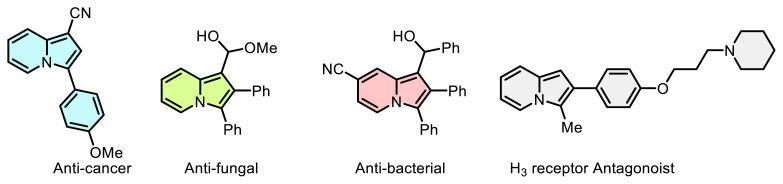
Some examples of pharmaceuticals featuring the indolizine motif.

On the other hand, the investigation of the synthesis of *N*-fused heterocyclic compounds synthesis is a growing and enduring domain within synthetic organic chemistry. Among these compounds, indolizines stand out as a significant class of heterocycles, and their chemistry has been comprehensively reviewed [23,24]. The indolizine framework has consistently captivated researchers due to its intriguing and structurally complex nature as a nitrogen heterocyclic moiety. Indeed, numerous indolizine derivatives have been discovered to display diverse biological activities. These include inhibition of phosphatase and aromatase enzymes [25,26], antibacterial effects against mycobacterium tuberculosis [27], antioxidant properties [28], antagonism of 5-hydroxytryptamine (5-HT_3_) receptors [29], calcium entry-blocking capabilities [30], as well as exhibiting antileukemic and antidepressant activities (Scheme 1) [31,32]. The biological and medicinal significance of indolizine derivatives has prompted the demand for effective synthetic methodologies to obtain these compounds. Consequently, several systematic endeavors have been directed towards the development of such strategies [33]. In this study, a practical and rapid synthetic method of azoindolizine derivatives via aryldiazonium salts with excellent yields was reported.

## 2. Materials and methods

Nuclear magnetic resonance (NMR) experiments were conducted using Varian and Bruker Avance II instruments (400 MHz for ^1^H NMR and 100 MHz for ^13^C NMR analysis). The solvents used for NMR was DMSO-*d_6_* and acetone-*d_6_*. Chemical shifts are reported in parts per million (δ/ppm). Coupling constants are reported in hertz (J/Hz). The peak patterns are indicated as follows: singlet, s; doublet, d; triplet, t; quadruplet, q; multiplet, m; doublet of doublets, dd; and broad singlet, bs. Melting points were determined on Gallenkamp melting point apparatus. Aryl diazonium salts (2a–g) were prepared according to literature [34,35]. All NMR spectra were reported in the Supporting Information.

### General procedure 1(GP1): Preparation of aryl diazonium salts (2a–g)

Aniline (5 mmol) was dissolved in a mixture of absolute ethanol (3 mL) and an aqueous solution of HBF_4_ (50%, 1.25 mL, 10 mmol). After cooling the reaction mixture to 0 °C, *tert*-butylnitrite (1.40 mL, 10 mmol) was added dropwise. The resulting mixture was stirred at room temperature for 1 h. Diethyl ether (10 mL) was added to precipitate the aryl diazonium tetrafluoroborate. It was then filtered off and washed with diethyl ether (3×10 mL). The aryl diazonium tetrafluoroborates (2) were dried and directly used without further purification.

### Phenyldiazonium tetrafluoroborate (2a)

Following the GP1, 2a was obtained as a white solid (900 mg, 87%).^1^H NMR (400 MHz, DMSO-*d_6_*) δ 8.76–8.47 (m, 2H), 8.39–8.14 (m, 1H), 8.08–7.84 (m, 2H). ^13^C NMR (100 MHz, DMSO-*d_6_*) δ 140.8, 132.6, 131.2, 116.0.

### 4-Clorobenzenediazonium tetrafluoroborate (2b)

Following the GP1, 2b was obtained as a white solid (1.03 g, 89%).^1^H NMR (400 MHz, Acetone-*d_6_*) δ 8.88 (d, *J* = 9.1 Hz, 2H), 8.16 (d, *J* = 9.1 Hz, 2H). ^13^C NMR (100 MHz, Acetone-*d_6_*) δ 149.0, 135.4, 132.9, 115.0.

### 4-Bromobenzenediazonium tetrafluoroborate (2c)

Following the GP1, 2c was obtained as a white solid (1.2 g, 86%).^1^H NMR (400 MHz, Acetone-*d_6_*) δ 8.78 (d, *J* = 9.1 Hz, 2H), 8.33 (d, *J* = 9.1 Hz, 2H). ^13^C NMR (100 MHz, Acetone-*d_6_*) δ 138.5, 136.0, 135.0, 115.6.

### 4-Methylbenzenediazonium tetrafluoroborate (2d)

Following the GP1, 2d was obtained as a white solid (890 mg, 84%). ^1^H NMR (400 MHz, Acetone-*d_6_*) δ 8.71 (d, *J* = 8.7 Hz, 2H), 7.90 (d, *J* = 8.7 Hz, 2H), 2.69 (s, 3H). ^13^C NMR (100 MHz, Acetone-*d_6_*) δ 156.1, 133.7, 133.1, 112.5, 22.9.

### 3-Methylbenzenediazonium tetrafluoroborate (2e)

Following the GP1, 2e was obtained as a white solid (950 mg, 89%).^1^H NMR (400 MHz, DMSO-*d_6_*) δ 8.66–8.35 (m, 2H), 8.10 (d, *J* = 7.5 Hz, 1H), 7.87 (t, *J* = 7.5 Hz, 1H). 2.50 (s, 3H). ^13^C NMR (100 MHz, DMSO-*d_6_*) δ 141.7, 131.7, 131.0, 130.0, 129.1, 115.4, 20.5.

### 4-Methoxybenzenediazonium tetrafluoroborate (2f)

Following the GP1, 2f was obtained as a white solid (930 mg, 83%).^1^H NMR (400 MHz, DMSO-*d_6_*) δ 8.62 (d, *J* = 9.4 Hz, 2H), 7.49 (d, *J* = 9.4 Hz, 2H), 4.05 (s, 3H). ^13^C NMR (100 MHz, DMSO-*d_6_*) δ 168.8, 136.1, 117.2, 103.3, 57.4.

### 3-Methoxybenzenediazonium tetrafluoroborate (2g)

Following the GP1, 2g was obtained as a white solid (945 mg, 85%).^1^H NMR (400 MHz, DMSO-*d_6_*) δ 8.46–8.20 (m, 2H), 8.01–7.70 (m, 2H), 3.92 (s, 3H). ^13^C NMR (100 MHz, DMSO-*d_6_*) δ 159.5, 132.3, 128.0, 125.4, 116.5, 115.8, 56.6.

### General procedure 2 (GP2): Preparation of azoindolizine derivatives (3a–h)

To a solution of indolizine (0.25 mmol) in CH_2_Cl_2_ (3 mL), aryl diazonium tetrafluoroborate (0.25 mmol) was added and the mixture was stirred at room temperature for 5 min. After the reaction was complete the solvent was evaporated under reduced pressure. The residue was rinsed with diethyl ether to give the desired product.

### 2-Phenyl-3-(phenyldiazenyl)indolizine (3a)

Following the GP2, 3a was obtained as a red solid (75 mg, 98%; mp 195.5–196.5 °C). ^1^H NMR (400 MHz, DMSO-*d_6_*) δ 10.17 (d, *J* = 7.0 Hz, 1H), 8.03–7.91 (m, 2H), 7.81 (d, *J* = 8.7 Hz, 1H), 7.78–7.71 (m, 2H), 7.59–7.47 (m, 4H), 7.45–7.36 (m, 2H), 7.33 (t, *J* = 7.3 Hz, 1H), 7.24–7.12 (m, 2H). ^13^C NMR (100 MHz, DMSO-*d_6_*) δ 153.4, 136.6, 135.2, 133.6, 130.7, 129.8, 129.2, 128.5, 128.4, 127.91, 127.88, 126.3, 120.9, 119.0, 115.9, 104.9.

### 3-((4-Chlorophenyl)diazenyl)-2-phenylindolizine (3b)

Following the GP2, 3b was obtained as a red solid (82 mg, 95%; mp 199.4–200.4 °C). ^1^H NMR (400 MHz, DMSO-*d_6_*) δ 10.11 (d, *J* = 7.0 Hz, 1H), 7.98–7.85 (m, 2H), 7.77 (d, *J* = 8.7 Hz, 1H), 7.70 (d, *J* = 8.7 Hz, 2H), 7.54–7.45 (m, 4H), 7.44–7.33 (m, 2H), 7.19–7.09 (m, 2H). ^13^C NMR (100 MHz, DMSO-*d_6_*) δ 152.3, 136.8, 135.6, 133.5, 131.7, 130.6, 129.8, 129.2, 128.7, 128.4, 127.9, 126.4, 122.3, 119.0, 116.0, 105.1.

### 3-((4-Bromophenyl)diazenyl)-2-phenylindolizine (3c)

Following the GP2, 3c was obtained as a red solid (94 mg, 97%; mp 199.7–200.7 °C). ^1^H NMR (400 MHz, DMSO-*d_6_*) δ 10.19 (d, *J* = 6.9 Hz, 1H), 8.03–7.89 (m, 2H), 7.83 (d, *J* = 8.7 Hz, 1H), 7.75–7.64 (m, 4H), 7.53 (t, *J* = 7.5 Hz, 2H), 7.43 (t, *J* = 7.5 Hz, 2H), 7.29–7.11 (m, 2H). ^13^C NMR (100 MHz, DMSO-*d_6_*) δ 152.7, 136.9, 135.7, 133.5, 132.1, 130.7, 129.8, 128.7, 128.4, 127.9, 126.5, 122.7, 120.4, 119.0, 116.1, 105.1.

### 2-Phenyl-3-(*p*-tolyldiazenyl)indolizine (3d)

Following the GP2, 3d was obtained as a black solid (77 mg, 95%; mp 182.4–1183.4 °C). ^1^H NMR (400 MHz, DMSO-*d_6_*) δ 10.15 (d, *J* = 6.9 Hz, 1H), 8.05–7.91 (m, 2H), 7.79 (d, *J* = 8.5 Hz, 1H), 7.67 (d, *J* = 8.5 Hz, 2H), 7.52 (t, *J* = 7.6 Hz, 2H), 7.46–7.27 (m, 4H), 7.20–7.03 (m, 2H), 2.36 (s, 3H). ^13^C NMR (100 MHz, DMSO-*d_6_*) δ 151.3, 137.7, 136.3, 134.7, 133.6, 130.6, 129.8, 129.7, 128.4, 128.4, 127.8, 125.7, 120.8, 119.0, 115.7, 104.6, 20.8.

### 2-Phenyl-3-(*m*-tolyldiazenyl)indolizine (3e)

Following the GP2, 3e was obtained as a black solid (79 mg, 96%; mp 174.0–175.0 °C). ^1^H NMR (400 MHz, DMSO-*d_6_*) δ 10.18 (d, *J* = 7.0 Hz, 1H), 8.00–7.95 (m, 2H), 7.82 (d, *J* = 8.7 Hz, 1H), 7.62 (s, 1H), 7.59–7.50 (m, 3H), 7.47–7.35 (m, 3H), 7.22–7.11 (m, 3H), 2.40 (s, 3H). ^13^C NMR (100 MHz, DMSO-*d_6_*) δ 153.6, 138.5, 136.5, 135.1, 133.6, 130.6, 129.8, 129.0, 128.6, 128.5, 128.4, 127.9, 122.2 (2C), 119.0, 117.4, 115.9, 104.7, 21.1.

### 3-((4-Methoxyphenyl)diazenyl)-2-phenylindolizine (3f)

Following the GP2, 3f was obtained as a red solid (81 mg, 96%; mp 178.0–1179.0 °C). ^1^H NMR (400 MHz, DMSO-*d_6_*) δ 10.09 (d, *J* = 7.0 Hz, 1H), 7.95 (d, *J* = 7.3 Hz, 2H), 7.79–7.69 (m, 3H), 7.50 (t, *J* = 7.6 Hz, 2H), 7.39 (t, *J* = 7.3 Hz, 1H), 7.33–7.25 (m, 1H), 7.15–7.01 (m, 4H), 3.81 (s, 3H). ^13^C NMR (100 MHz, DMSO-*d_6_*) δ 159.5, 147.7, 135.6, 134.0, 133.8, 130.5, 129.8, 128.4, 128.1, 127.6, 124.9, 122.5, 118.9, 115.3, 114.5, 103.9, 55.4.

### 3-((3-Methoxyphenyl)diazenyl)-2-phenylindolizine (3g)

Following the GP2, 3g was obtained as a red solid (80 mg, 95%; mp 101.0–102.0 °C). ^1^H NMR (400 MHz, DMSO-*d_6_*) δ 10.14 (d, *J* = 6.9 Hz, 1H), 7.97 (d, *J* = 7.5 Hz, 2H), 7.79 (d, *J* = 8.7 Hz, 1H), 7.51 (t, *J* = 7.5 Hz, 2H), 7.45–7.31 (m, 5H), 7.23–7.11 (m, 2H), 6.96–6.84 (m, 1H), 3.82 (s, 3H). ^13^C NMR (100 MHz, DMSO-*d_6_*) δ 160.1, 154.9, 136.7, 135.4, 133.5, 130.4, 129.9, 129.8, 128.7, 128.4, 127.9, 126.3, 119.0, 116.0, 114.6, 114.2, 104.4, 104.3, 55.0.

### 3-(Phenyldiazenyl)-2-(*p*-tolyl)indolizine (3h)

Following the GP2, 3h was obtained as a red solid (74 g, 94%; mp 173.5–174.5 °C). ^1^H NMR (400 MHz, DMSO-*d_6_*) δ 10.11 (d, *J* = 7.0 Hz, 1H), 7.83 (d, *J* = 8.0 Hz, 2H), 7.76–7.70 (m, 3H), 7.46 (t, *J* = 7.8 Hz, 2H), 7.37–7.26 (m, 4H), 7.16–7.08 (m, 2H), 2.34 (s, 3H). ^13^C NMR (100 MHz, DMSO-*d_6_*) δ 154.1, 138.1, 137.5, 136.1, 131.4, 130.3, 129.9, 129.8, 129.3, 128.8, 128.5, 127.0, 121.5, 119.6, 116.5, 105.3, 21.5.

## 3. Results

Initially, aryl diazonium salts were synthesized from the corresponding aniline derivatives with good yields. The study commenced by using indolizine and phenyldiazonium tetrafluoroborate salt as standard substrates to investigate the optimal reaction conditions (Table). The impact of solvents on the reaction yield was assessed. Various protic solvents, including H_2_O, EtOH, and MeOH, were tested, resulting in the desired product 3a being obtained in yields ranging from 75% to 77% (Table, entries 1–3). When various aprotic solvents, including THF, CH_3_CN, DMF, DMSO, and CH_2_Cl_2_, were employed, product 3a was achieved with yields ranging from 80% to 95% (Table, entries 4–8). Notably, aprotic solvents were observed to be considerably more favorable than protic solvents, with CH_2_Cl_2_ yielding the highest results. Following the completion of screenings, these optimized conditions were applied, which consisted of 0.25 mmol of indolizine (1), 0.25 mmol of phenyldiazonium tetrafluoroborate salt (2), room temperature (rt), a 5-min reaction time, and CH_2_Cl_2_ as the solvent. This allowed us to broaden the applicability of this method to various sources of indolizine and phenyldiazonium tetrafluoroborate salt.

**Scheme 2:**
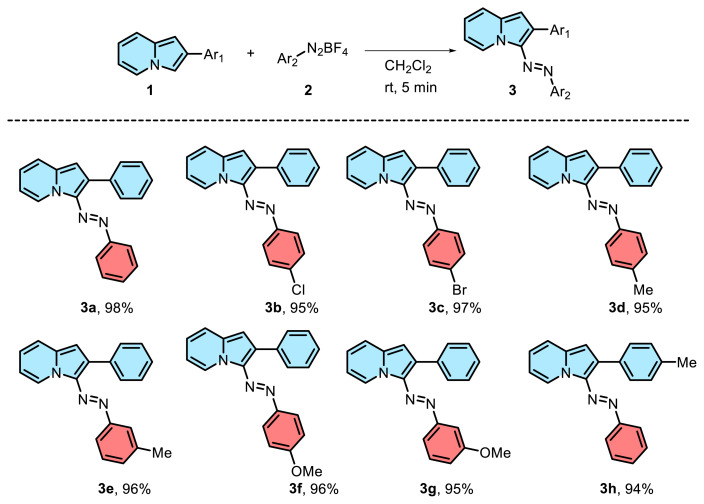
Substrate scope study.

Having successfully established the optimized reaction conditions, the subsequent step was to investigate the reaction’s applicability to a range of diazonium tetrafluoroborate salts (Scheme 2). Initially, the reaction was assessed using various substituents on the phenyl ring of the diazonium tetrafluoroborate salt. For this purpose, indolizine (1) was treated with different diazonium tetrafluoroborate salts 2a–g. The reaction of indolizines (1) with *p*-halogenated diazonium tetrafluoroborate salts were transformed into the corresponding products 3b–3c with excellent yields of 95% and 97% respectively. Delightedly, substituted diazonium tetrafluoroborate salts with electron-donating groups, whether the substituents are at meta-, or *para*-position, afforded the corresponding products 3d–3g in high yields (94%–96%). Next, the applicability of indolizine was evaluated. As expected, the reaction of 2-(*p*-tolyl)indolizine with phenyldiazonium tetrafluoroborate salt was established as the desired product 3h in excellent yield (94%).

## 4. Discussion

In this context, a practical, rapid, and efficient method for the synthesis of azoindolizine derivatives via aryldiazonium salts was successfully developed. Through systematic optimization of reaction conditions, achieved excellent yields (up to 98%), demonstrating the robustness of the approach. Additionally, the scope of this method to various sources of indolizine and aryldiazonium tetrafluoroborate salt, enhancing its applicability was expanded.

## Figures and Tables

**Table:** Solvent optimization study of the formation of 3a.

Entry	Solvent	Yield (%)
1	H_2_O	77
2	EtOH	75
3	MeOH	76
4	THF	85
5	CH_3_CN	83
6	DMF	80
7	DMSO	90
**8**	**CH** **_2_** **Cl** **_2_**	**95**
